# Cost-effectiveness of internet-based cognitive–behavioural therapy and physical exercise for depression

**DOI:** 10.1192/bjo.2018.38

**Published:** 2018-07-16

**Authors:** Martin Kraepelien, Simon Mattsson, Erik Hedman-Lagerlöf, Ingemar F. Petersson, Yvonne Forsell, Nils Lindefors, Viktor Kaldo

**Affiliations:** Department of Clinical Neuroscience, Centre for Psychiatry Research, Karolinska Institutet, Sweden; Department of Clinical Neuroscience, Division of Psychology, Karolinska Institutet, Sweden; Department of Clinical Neuroscience, Division of Psychology, Karolinska Institutet, Sweden; Department of Clinical Neuroscience, Osher Center for Integrative Medicine, Karolinska Institutet, Sweden; Department of Clinical Sciences, Department of Orthopaedics, Lund University, Sweden; Department of Public Health Sciences, Section for Epidemiology and Public Health Intervention Research, Karolinska Institutet, Sweden; Department of Clinical Neuroscience, Centre for Psychiatry Research, Karolinska Institutet, Sweden; Department of Clinical Neuroscience, Centre for Psychiatry Research, Karolinska Institutet, Sweden; Department of Psychology, Faculty of Health and Life Sciences, Linnaeus University, Sweden

**Keywords:** Cognitive–behavioural therapies, cost-effectiveness, depressive disorders, randomised controlled trial, exercise

## Abstract

**Background:**

Both internet-based cognitive–behavioural therapy (ICBT) and physical exercise are alternatives to treatment as usual (TAU) in managing mild to moderate depression in primary care.

**Aims:**

To determine the cost-effectiveness of ICBT and physical exercise compared with TAU in primary care.

**Method:**

Economic evaluation of a randomised controlled trial (*N* = 945) in Sweden. Costs were estimated by a service use questionnaire and used together with the effects on quality-adjusted life-years (QALYs). The primary 3-month healthcare provider perspective in primary care was complemented by a 1-year societal perspective.

**Results:**

The primary analysis showed that incremental cost per QALY gain was €8817 for ICBT and €14 571 for physical exercise compared with TAU. At the established willingness-to-pay threshold of €21 536 (£20 000) per QALY, the probability of ICBT being cost-effective is 90%, and for physical exercise is 76%, compared with TAU.

**Conclusions:**

From a primary care perspective, both ICBT and physical exercise for depression are likely to be cost-effective compared with TAU.

**Declaration of interest:**

None.

Depression is projected by the World Health Organization to be the leading cause of disease burden worldwide by 2030.^1^ In addition to the effects of depression on the individual's mortality, disability and quality of life, there are high societal costs. These costs consist of healthcare costs (direct costs) such as healthcare procedures and medications, and societal non-healthcare costs (indirect costs) such as loss of production.^2^ Therefore, new effective treatments for depression that can be shown to be associated with less costs than usual care are needed. Some smaller (*n* = 263–297) previous studies have found internet-based treatments for depression to be cost-effective.^3^^–^^6^ There is also evidence that physical exercise interventions are cost-effective in specific settings and for specific population groups, but more studies in primary care settings are needed.^7^^,^^8^ Additionally, larger cost-effectiveness analyses of both internet-based treatments and physical exercise in the treatment of depression in primary care are lacking. A recently conducted randomised controlled trial (the Regassa study^9^^–^^11^) found both individually tailored internet-based cognitive–behavioural therapy (ICBT) and monitored physical exercise to be more effective in reducing symptoms of depression and improving psychological functioning and sleep compared with treatment as usual (TAU) in primary care after 12 months. No significant differences were seen between the ICBT and physical exercise groups. Our aim was to investigate the cost-effectiveness of ICBT and physical exercise compared with TAU, using data from this large-scale randomised trial.

## Method

This cost-effectiveness study was conducted alongside a multi-site (primary care centres in six county councils in Sweden; the analysis did not consider eventual clustering effects), randomised controlled trial of ICBT and physical exercise compared with TAU for primary care patients with depression. The trial was registered with the registry of clinical trials in Stockholm county (Karolinska Clinical Trials Registry identifier: KT20110063) and approved by the Regional Ethics Review Board in Stockholm, Sweden (Registration number 2010/1779-31/4). Participants were recruited between February 2011 and December 2012. The inclusion criteria were aged 18–67 years and present depressive symptoms defined as scoring ≥10 on the Patient Health Questionnaire (PHQ-9).^12^ A PHQ-9-score of 10 or above has both high sensitivity and specificity for major depression. Exclusion criteria were a need for specialist psychiatric treatment, substance dependency and not being able to understand Swedish. Participants (*N* = 945) were included and randomised in equal proportions to the three treatment groups. For more details on the sample and trial methods, see the earlier reports.^9^^,^^11^ All 945 participants were included in the analyses, in line with the principle of intention to treat.

The primary perspective for this economic evaluation was that of the healthcare provider in primary care during the 3-month intervention period. This included the costs of the ICBT and physical exercise interventions plus the following healthcare provider costs: visits to a general practitioner, social worker, physiotherapist or occupational therapist, and costs for other psychological treatments. To further explore this perspective, 1-year primary healthcare costs were also explored. The secondary perspective was that of 1-year societal costs, which included healthcare costs, direct non-medical costs (e.g. using self-help groups such as Alcoholics Anonymous, home help or seeing an alternative practitioner such as an acupuncturist) and societal non-healthcare costs (i.e. costs of unemployment, sick leave, productivity loss at work and productivity loss in the domestic realm) during 1 year from treatment initiation. Costs were not discounted because of the follow-up period of only 1 year.

### Interventions

The treatment period lasted 12 weeks and participants were randomised to one of three interventions: ICBT, physical exercise and TAU in primary care. Both ICBT and physical exercise were additions to TAU in the sense that participants in these arms were still allowed to utilise primary care services.

The ICBT intervention was similar to the internet-based treatment for depression used in routine psychiatric care in Stockholm County, Sweden,^13^ but was based on an individually tailored approach also used in earlier ICBT for populations with high comorbidity.^14^ This type of ICBT is based on self-help texts similar to chapters in cognitive–behavioural therapy self-help books, combined with practical homework assignments each week. The treatment included a mandatory component of behavioural activation for depression^15^ and was then tailored to the individual with other modules based on cognitive–behavioural therapy for different comorbid conditions such as worry, panic, social anxiety, stress, insomnia and pain. Therapist support was given as written messages in the treatment platform. The therapists were licensed clinical psychologists or psychology students supervised by a licensed psychologist.

The physical exercise intervention consisted of a maximum of 36 supervised group exercise sessions during the treatment period (three times a week), a weekly meeting with a trainer or physiotherapist and phone calls to monitor adherence and encourage inactive participants to carry on with the exercise programme. Phone reminders were scheduled once a week, if needed. Several attempts to contact the participant were made if there was no answer.

TAU consisted of the treatment the participant received at their primary care unit, determined by the participant's primary care physician. In many cases this included counselling, but not all participants received any depression treatment at their primary care unit. The magnitude of the treatment administered by the primary care unit, before adding the intervention costs of ICBT and physical exercise, is reflected in the cost table of the healthcare provider perspective (Table 2).

### Utility and depression assessment

Utility was measured with quality-adjusted life-years (QALYs). QALYs were calculated based on the self-rated health-related quality-of-life measure EuroQol-5D-3L (EQ-5D^16^), using the UK weights,^17^ which are used as standard in Stockholm county, to value responses in five health-related areas of life. The EQ-5D was administered as a paper questionnaire with interviewer support at baseline, and self-administered questionnaire post-treatment and at the 12-month follow-up. The area under the curve approach was used between the three time points to calculate the QALYs produced during the 12 months after treatment initiation. In other words, utility values from each person's EQ-5D measurements were multiplied by the length of time spent in that state to construct one QALY score per person. A QALY score of 1.0 would indicate the maximum score, and the highest possible health-related quality of life during the 12-month period.

Montgomery–Åsberg Depression Rating Scale (MADRS^18^) was administered by a trained research nurse at all three time points, either face-to-face or by telephone. Response on the MADRS was used as a secondary effect measure and a responder was defined as a participant with a depression severity score more than 1 s.d. below the baseline group average at the 12-month follow-up, as used in the earlier reported results on depression.^11^

### Cost assessment

The Trimbos and iMTA questionnaire on Costs associated with Psychiatric illness (TiC-P^19^) was used as a self-administered paper questionnaire to collect cost data. The TiC-P has shown good reliability and satisfactory validity when compared with registry data.^20^ The parts of TiC-P estimating healthcare resource use (e.g. seeing a general practitioner or social worker) were collected at baseline, 3 months and 12 months after baseline. Parts of TiC-P estimating direct non-medical costs and societal non-healthcare costs were collected at baseline and at 12 months after baseline. Only the costs of healthcare resource use in primary care collected at the 3-month time point were included in the healthcare perspective. All costs from the entire 12-month period were included in the societal perspective. The TiC-P covered costs during the previous month at baseline and at the 12-month time point, which was then extrapolated to the relevant time period. The service use questions at the 3-month time point covered costs during the previous 3 months. A 1-year healthcare provider perspective, consisting of only primary healthcare costs but collected during the full 12-month period, was explored as an additional analysis.

The costs were assessed in Swedish Krona (SEK) and converted to Euro (€), using purchasing power parities and 2012 as reference year (1 SEK = € 0.08729627^21^). The cost tariffs of healthcare services were obtained from the official indexes of the county councils for services offered within the publicly funded healthcare system in Sweden. The costs of medications were collected from the webpage of Sweden's state-owned pharmacy (www.apoteket.se). The participants' gender and profession were used together with national average data from the Swedish Central Bureau of Statistics to calculate each participants' predicted salary. The salary was combined with the self-reported amount of productivity loss at work, unemployment and sick leave reported in TiC-P to estimate corresponding societal cost items. Productivity losses in the domestic realm were valued reflecting the market price of domestic help in Sweden. The intervention cost of ICBT was calculated based on the tariffs for a licensed psychologist plus overhead costs (e.g. rents, administration) multiplied with time spent treating patients. The intervention cost of physical exercise was correspondingly calculated by multiplying the salary of gym leaders, physiotherapists and research nurses, plus overhead costs, with the time spent on sessions and phone call reminders. The gym leader was conservatively presumed to be leading classes with 20 participants, and to lead the class even if some individual participants were missing.

### Cost-effectiveness analysis

The primary cost-effectiveness analysis was the cost–utility analysis in the healthcare provider perspective. A cost–utility analysis compares the difference in produced QALYs between two treatment alternatives with the difference in costs generated. The cost–utility analysis was also performed in the 1-year societal perspective. Secondary cost-effectiveness analyses compared response on depression with the difference in costs in the healthcare and 1-year societal perspective.

Incremental cost-effectiveness ratios (ICERs) were computed as the difference in costs between two treatment alternatives divided by the difference in effects between the same two treatments. Because the differences in effects seen between ICBT and physical exercise were negligible,^9^^,^^11^ and values close to 0 in the denominator would result in an inflated and incomprehensible ICER, the cost-effectiveness analysis was applied to ICBT compared with TAU and to physical exercise compared with TAU, but not directly between ICBT and physical exercise.

A treatment is usually considered cost-effective if it is both more effective and less expensive than the comparison treatment, or if the calculated costs per extra unit of improvement are less than the society's willingness to pay (WTP) for each unit of improvement. In this analysis we used the commonly used UK National Institute for Health and Care Excellence WTP threshold of £20 000 (€21 536, using the earlier mentioned 2012 purchasing power parities for conversion) per QALY^22^ to indicate a cost-effective treatment in the healthcare perspective. Alternative WTP thresholds were also explored in both perspectives with cost-effectiveness acceptability curves (CEACs). There is no established Swedish WTP threshold, but for a secondary interpretation within the Swedish context, we used national guidelines for cardiac care that mention preliminary cut-offs for costs per QALY. Below SEK 100 000 (€8730) was considered as low costs per QALY and below SEK 500 000 (€43 648) was considered as moderate costs per QALY in these guidelines.^23^

### Statistical analysis

Statistical analyses were conducted in R version 3.3.1 (R Core team) and SPSS version 23 (IBM Corp). Of the available EQ-5D questionnaires used to calculate QALYs, 25% were missing at post-treatment and 17% were missing at 1-year follow-up. For MADRS, 22% were missing at post-treatment and 15% were missing at 1-year follow-up. Four participants (<1%) of TiC-P questionnaires were missing at baseline, 23% were missing at post-treatment and 40% were missing at 1-year follow-up. Some missing TiC-P items at the 1-year follow-up corresponded to items from other concurrent questionnaires, bringing the amount of missing data for sick leave and unemployment down to 15%.

Remaining missing data were then imputed with multiple imputation by chained equations, using probability mean matching, to be used in the cost-effectiveness analyses. The missing data was imputed separately by randomised group and were assumed to be missing at random. The number of imputations were set to 40 to exceed the percentage of missing data, following the procedure in White *et al*.^24^ The imputation model used demographic variables (gender, age, work status and education level), baseline self-reported work ability and the relevant outcome at baseline for imputing costs and effects, respectively. Auxiliary variables were included in the imputation model based on their theoretically potential relation to both the values and missingness status of the outcome variables. Effects and costs in the healthcare perspective were imputed at the level of individual items. Societal costs, however, were imputed at the aggregated level (e.g. total cost of sick-leave). Rubin's rules^25^ were used to calculate means from the imputed data-set for constructing ICER point estimates. Imputed costs and effects are presented with pooled s.d.

To account for the uncertainty of the ICER point estimates, 5000 ICERs per imputed data-set were simulated by Monte Carlo simulating with non-parametric bootstrapping. These simulated ICERs were used to construct the CEACs to estimate the probabilities of the treatment alternative being cost-effective compared with TAU at different WTP thresholds in both the healthcare and societal perspectives. The probabilities of the treatment alternative being cost-effective compared with TAU at the chosen WTP threshold of €21 536 in the healthcare perspective were also presented.

## Results

**Fig. 1:**
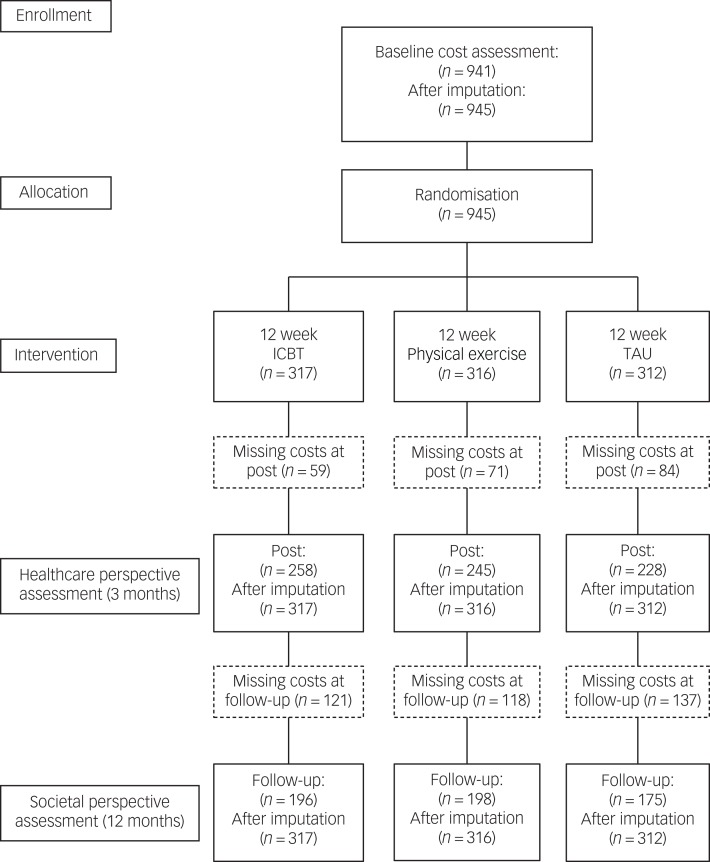
Participants' flow through the study. ICBT, internet-based cognitive–behavioural therapy; TAU, treatment as usual.

Of the 945 participants included in this analysis, 317 were randomised to ICBT, 316 to physical exercise and 312 to the TAU condition, and are presented in the participant flow chart (Fig. 1). The participants were predominantly women (73%), born in Sweden (80%) and the mean age was 43 years. Comorbid depression and anxiety were common (67%) and equally many reported moderate to severe physical pain (68%).^9^ Participant characteristics are shown in Table 1.

**Table 1:** Baseline demographics

	ICBT, *n* = 317	Physical exercise, *n* = 316	TAU, *n* = 312
Age			
Mean (95% CI)	43.2 (41.8–44.6)	42.6 (41.3–43.9)	43.2 (41.9–44.6)
Gender			
Female, *n* (%)	229 (72.2)	220 (69.6)	240 (76.9)
Marital status			
Married or cohabiting, *n* (%)	149 (47.0)	140 (44.3)	153 (49.0)
Employment status			
Unemployed, *n* (%)	44 (13.9)	39 (12.3)	36 (11.5)
Country of birth			
Sweden, *n* (%)	253 (79.8)	249 (78.8)	249 (79.8)
Education, *n* (%)			
Primary school	9 (2.8)	15 (4.7)	11 (3.5)
Technical school	39 (12.3)	40 (12.7)	33 (10.6)
Secondary school	71 (22.4)	79 (25.0)	75 (24.0)
Post-secondary	62 (19.6)	52 (16.5)	62 (19.9)
Tertiary	135 (42.6)	129 (40.8)	128 (41.0)

ICBT, internet-based cognitive–behavioural therapy; TAU, treatment as usual.

### Utility and depression

Both ICBT and physical exercise were associated with slightly more produced QALYs and a higher proportion of treatment responders than TAU, at the 12-month follow-up (Table 2).

**Table 2:** Effects on imputed utility and depression scores at 12-month follow-up

	*N*	QALYs		Responders	
		Mean	(s.e.)	Mean proportion	(s.e.)
ICBT	317	0.6909	(0.1037)	0.7631	(0.1551)
Physical exercise	316	0.6905	(0.1024)	0.7498	(0.1565)
TAU	312	0.6571	(0.1085)	0.6819	(0.1632)

ICBT, internet-based cognitive–behavioural therapy; QALYs, quality-adjusted life-years; Responders, proportion of participants with a Montgomery–Åsberg Depression Rating Scale score 1 s.d. lower or more than the baseline mean at 12-month follow-up; TAU, treatment as usual.

### Intervention attendance rates

In ICBT, the psychologist spent an average of 194 min with each participant (95% CI 175–212), which equals around 16 min of therapist time per participant per treatment week. In the physical exercise group, participants attended an average of 14.59 (95% CI 12.62–16.56) 1-hour classes out of 36 possible, 4.86 (95% CI 4.21–5.52) physiotherapist sessions out of 12 and were reminded by phone an average of 7.14 (95% CI 6.48–7.79) times.

### Costs: 3-month healthcare provider perspective

Because of their higher service use, especially a higher use of psychological treatment, participants in the TAU group incurred the highest costs of healthcare use during the treatment period. When adding the intervention costs of ICBT and physical exercise, the observed costs were slightly higher in the physical exercise group than in the ICBT and TAU groups (Tables 3 and 4).

**Table 3:** Imputed resource use during the 3-month intervention period: primary healthcare provider perspective

	ICBT		Physical exercise		TAU	
	Mean visits	(s.e.)	Mean visits	(s.e.)	Mean visits	(s.e.)
General practitioner	1.32	(0.04)	1.60	(0.07)	1.67	(0.09)
Social worker	0.26	(0.04)	0.24	(0.05)	0.71	(0.10)
Physiotherapist/occupational therapist	0.95	(0.08)	0.62	(0.07)	1.71	(0.15)

	Received treatment, %	(s.e.)	Received treatment, %	(s.e.)	Received treatment, %	(s.e.)
Psychotherapy/psychological treatment						
Counselling	5.88	(0.94)	13.18	(1.27)	20.38	(1.49)
Individual CBT	3.47	(0.62)	3.43	(0.83)	8.58	(1.20)
Group CBT	0.79	(0.50)	0.69	(0.53)	0.81	(0.54)
Psychodynamic therapy	0.71	(0.35)	1.26	(0.49)	2.12	(0.74)
Unguided self-help/internet treatment	1.87	(0.65)	0.79	(0.51)	1.72	(0.64)
Other psychotherapy/psychological treatment	5.11	(1.32)	6.09	(1.48)	7.54	(1.55)

CBT, cognitive–behavioural therapy; ICBT, internet-based cognitive–behavioural therapy; TAU, treatment as usual.

**Table 4:** Imputed costs (€) during the 3-month intervention period: primary healthcare provider perspective

	ICBT		Physical exercise		TAU	
	Mean cost	(s.e.)	Mean cost	(s.e.)	Mean cost	(s.e.)
General practitioner	192.14	(7.89)	230.73	(10.42)	241.39	(13.03)
Social worker	18.97	(3.16)	17.64	(3.94)	51.38	(5.89)
Physiotherapist/occupational therapist	42.57	(3.65)	27.34	(2.89)	76.67	(7.23)
Psychotherapy/psychological treatment	56.01	(5.28)	79.42	(7.08)	143.81	(8.90)
Total 3-month primary healthcare costs	309.69	(10.92)	355.13	(15.43)	513.25	(20.68)
Intervention costs	501.08	(5.03)	644.14	(10.75)	–	–
Total costs	810.77	(11.51)	999.27	(18.74)	513.25	(20.68)

ICBT, internet-based cognitive–behavioural therapy; TAU, treatment as usual.

### Costs: additional 1-year healthcare provider perspective

The imputed 12-month healthcare provider costs (s.e.) were €1753 (81) for ICBT, €2187 (154) for physical exercise and €1911 (128) for TAU. When adding the intervention costs, both ICBT and physical exercise became more expensive than TAU in the 12-month healthcare perspective.

### Costs: 1-year societal perspective

Costs were similar between groups in the 1-year societal perspective before adding the costs of treatment. The three largest costs in all three groups were related to unemployment, sick leave and healthcare use. When adding the costs of treatment, the observed costs of ICBT and physical exercise were slightly higher than TAU (Table 5).

**Table 5:** Imputed costs (€) during 1-year follow-up period: societal perspective

	ICBT		Physical exercise		TAU	
	Mean cost	(s.e.)	Mean cost	(s.e.)	Mean cost	(s.e.)
Healthcare costs	2747.50	(231.71)	3010.18	(170.25)	2276.48	(107.93)
Medications	27.86	(3.46)	39.26	(5.10)	39.11	(5.10)
Direct non-medical costs	329.56	(57.31)	254.34	(32.82)	306.37	(49.91)
Unemployment	4809.06	(398.42)	4614.58	(611.99)	4496.40	(707.03)
Sick leave^a^	2129.54	(377.22)	2012.11	(153.13)	1626.46	(222.31)
Productivity loss at work^b^	674.36	(71.96)	774.16	(86.79)	1476.41	(216.91)
Domestic productivity loss	466.16	(50.07)	541.05	(45.08)	402.01	(27.71)
Total indirect non-medical costs	8079.12	(613.11)	7941.91	(643.17)	8001.27	(760.67)
Total 1-year societal costs	11 184.03	(586.75)	11 245.69	(730.60)	10 623.23	(809.91)
Total including intervention costs	11 685.11	(586.75)	11 889.83	(731.12)	10 623.23	(809.91)

ICBT, internet-based cognitive–behavioural therapy; TAU, treatment as usual.

a. Societal costs of paid sick leave.

b. Calculated societal costs of lost productivity because of sickness at work.

### Cost-effectiveness

**Fig. 2:**
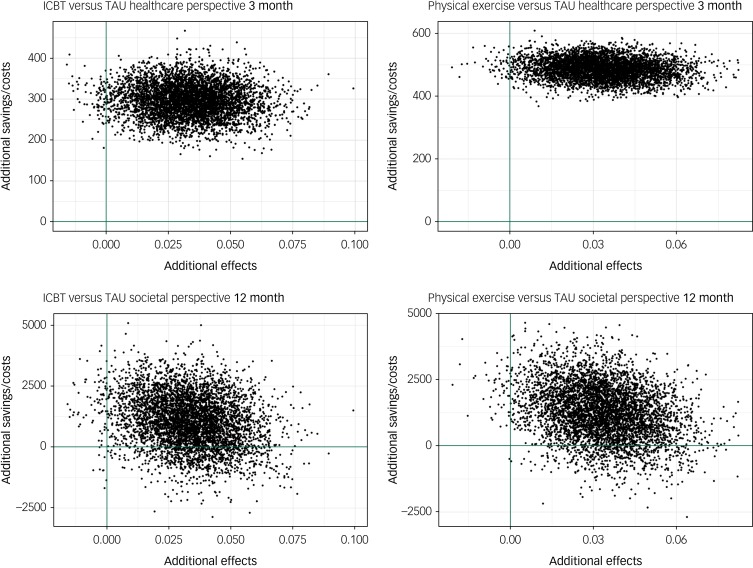
Cost-effectiveness utility planes presenting the scatter of 5000 bootstrapped incremental cost-effectiveness ratios. All costs are in Euros and effects are in quality-adjusted life-years. ICBT, internet-based cognitive–behavioural therapy; TAU, treatment as usual.

**Fig. 3:**
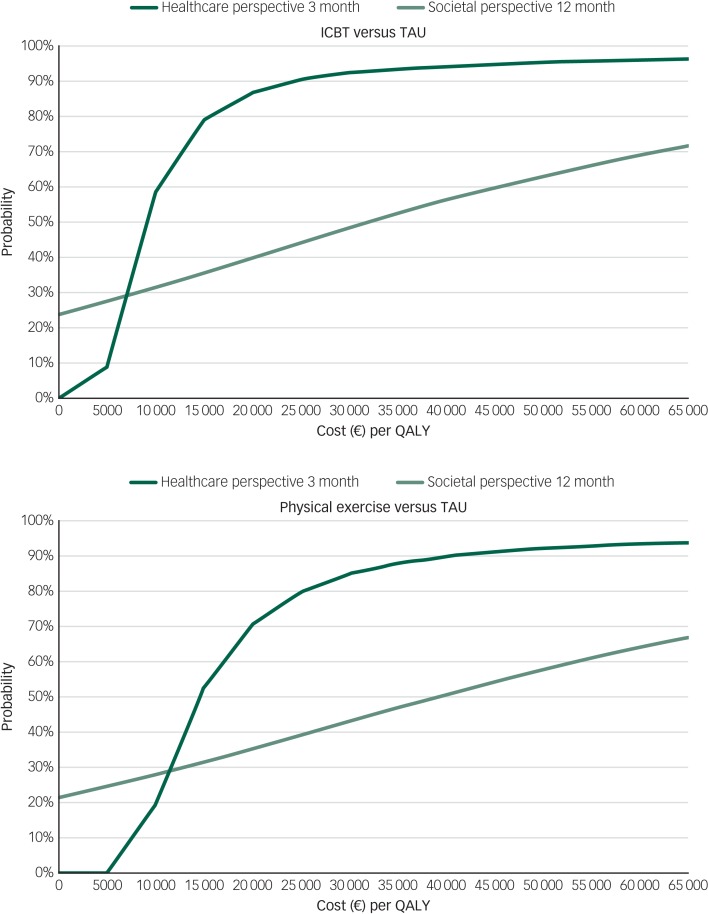
Cost-effectiveness acceptability curves illustrating the probability that ICBT (top) and physical exercise (bottom) is cost-effective compared with TAU at different levels of willingness to pay, in the healthcare and societal perspectives. ICBT, internet-based cognitive–behavioural therapy; QALY, quality-adjusted life-year; TAU, treatment as usual.

In the healthcare provider perspective base–case analysis, the cost difference between ICBT and TAU rendered a low ICER of €8817 per QALY, indicating that the treatment was cost-effective compared with TAU. The corresponding ICER based on the difference between physical exercise and TAU was €14 571 per QALY. The scatterplots with bootstrapped ICERs that represents the uncertainty around the ICER point estimates are shown in Fig. 2. As can be seen in the CEAC graphs (Fig. 3), both treatments become probably cost-effective with rising WTP thresholds. ICBT has a probability of 0.90 and physical exercise has a probability of 0.76 of being cost-effective at the chosen WTP threshold of €21 536 in the healthcare perspective. The larger differences in costs in the base–case societal perspective rendered higher ICERs of €31 471 for ICBT and €37 974 for physical exercise, compared with TAU.

In the additional 1-year healthcare provider perspective, the cost differences rendered an ICER of €10 166 per QALY for ICBT compared with TAU and €27 560 per QALY for physical exercise compared with TAU. ICBT has a probability of 0.83 and physical exercise has a probability of 0.32 of being cost-effective at a WTP threshold of €21 536, when considering the healthcare perspective for the full 12-month period.

The cost of one additional responder in depression was lower: €3666 for ICBT and €7157 for physical exercise in the 3-month healthcare provider perspective. In the 1-year societal perspective, the incremental costs per responder was €13 084 for ICBT and €18 652 for physical exercise, compared with TAU.

## Discussion

### Main findings

To our knowledge, this study is the largest prospective cost-effectiveness analyses of ICBT and physical exercise for mild to moderate depression. As seen in earlier reports from the Regassa study, ICBT, physical exercise and TAU all yielded a high proportion of treatment responders (defined as 1 s.d. lower or more than the baseline mean) at 12-month follow-up (range 67–77%), but the experimental treatments were slightly more effective than TAU. ICBT and physical exercise also produced slightly more QALYs than TAU. The TAU group used more resources in the 3-month treatment period than ICBT and physical exercise. However, when adding the extra costs of the interventions, ICBT and physical exercise turned out to be more expensive than TAU. When also adding the full 1-year societal perspective, ICBT and physical exercise both became even more expensive than TAU. However, the uncertainty around the cost estimates also increased.

ICBT and physical exercise seem to be cost-effective, compared with TAU, at the chosen WTP threshold of €21 536 per QALY, in the 3-month healthcare perspective. In the 1-year healthcare perspective, ICBT but not physical exercise is cost-effective compared with TAU at a WTP threshold of €21 536 per QALY. At a higher WTP threshold of €32 304 (£30 000) per QALY, the probability of ICBT is 0.91 and physical exercise is 0.60 to be cost-effective compared with TAU. When adding the full 1-year societal perspective, the difference in costs to TAU increased and the cost estimates tended to be associated with more uncertainty, which led to a need for WTP thresholds of around €30 000–40 000 to deem ICBT and physical exercise probable to be cost-effective compared with TAU in this setting. The costs per QALY are in the moderate range when compared with the earlier mentioned Swedish cut-offs for both ICBT and physical exercise compared with TAU, in all mentioned cost perspectives.^23^

### Comparison to prior studies: ICBT

The cost-effectiveness of ICBT in the treatment of depression is promising, especially when the treatment has been therapist-guided.^26^ In an early cost-effectiveness study of ICBT versus TAU, McCrone *et al*^3^ found ICBT for depression to be cost-effective at similar prices per QALY as in this study. The difference in produced QALYs between the treatments in that study was also very similar to this study; however, the QALYs were calculated indirectly and not based on the EQ-5D. The full societal perspective assessed by the TiC-P questionnaire was used by Warmerdam *et al*,^4^ who saw an ICER of €22 609, which is lower than the societal ICER in this study. However, the Warmerdam trial only compared the ICBT intervention to a wait-list condition and not an active treatment. Hollinghurst *et al*^5^ used QALYs based on the EQ-5D, and added loss of work to the cost perspective. The ICER were below €21 536 but the amount of missing cost data was higher than in this study, increasing risk for bias. The cost-effectiveness trial of an ICBT programme by Romero-Sanchiz *et al*^6^ produced a low cost per QALY of under €500 compared with TAU in the societal perspective; however, no intervention costs were added in this analysis, which is not a realistic assumption as a base–case analysis because it exaggerates the cost-effectiveness.

### Comparison to prior studies: physical exercise

Some studies have found physical exercise to be a cost-effective treatment but in quite specific settings or with specific subgroups.^8^ An example of this is a large trial by Edwards *et al*,^7^ who found an intervention with access to sessions to be cost-effective in a population with coronary heart disease risk factors. The incremental cost per QALY in the healthcare perspective was €13 041 (£12 111) in that trial, which is similar to the €14 571 per QALY gained found in our trial for the physical exercise intervention. One trial by Chalder *et al*,^27^ of a physical activity intervention in the treatment of depression in a more general setting, did not find the intervention to be cost-effective compared with TAU alone. That intervention, however, did not have supervised group exercise sessions, which could be an important property of the physical exercise intervention in this study. Another difference to our study is that the Chalder trial used the five-level and not the three-level version of EQ-5D.

### Strengths and limitations

Major strengths of this study were the randomised controlled design yielding experimental control of treatment effects, the large samples sizes in each treatment condition and the associated high statistical power, the analytic method which allowed for cost assessment from both a healthcare provider and societal perspective, and the use of primary care TAU and not wait-list as a comparator.

A limitation of the study was that we did not use registry data to collect data regarding costs, but relied on the self-reported TiC-P to this end. Previous research has however shown that the TiC-P has good agreement with registry data,^20^ indicating that cost estimates of the present study were valid. Another limitation of the study was that the estimation of costs for ICBT and physical exercise are associated with some degree of uncertainty, as they are dependent on the treatment setting. That is, in healthcare systems where the wages of, for example, psychologists and physiotherapists markedly differ from Swedish wages, the cost of the experimental treatments would differ too. As a means to deal with this issue, our study used conservative (high) estimates of intervention costs of ICBT and physical exercise. The time spent by the psychologist on each participant in the ICBT intervention in this study was also high (194 *v.* 149 min) compared with similar ICBT for depression in routine care, leading to conservative cost estimates. The cost of physical exercise is also conservatively estimated because higher attendance rates in physical exercise would not make the intervention costlier, as the leader was presumed to hold the class even with low attendance.

### Implications for policy and future research

This study showed that ICBT for depression is probably cost-effective, with a small cost per QALY. This means that, compared with primary care TAU, ICBT implemented as it was used in this trial can increase quality of life and reduce depressive symptoms at a small additional cost for the primary care unit. Together with the previously conducted research on clinical- and cost-effectiveness of ICBT, this study indicates that from the healthcare provider perspective, ICBT should be implemented in primary care. However, there are primary care studies showing no additional effects of ICBT.^28^^,^^29^ These did not use the current model with individually tailored ICBT and a centralised treatment unit specialised on ICBT, which points to the possible importance on how ICBT is implemented in routine care.

Importantly, this study showed that ICBT reduced visits to general practitioners and psychologists/psychiatrists, which suggest that offering ICBT leads to less strain on other healthcare resources. In Sweden, the infrastructure for distributing ICBT in routine care is rather well developed. At the same time, in many other countries the initial costs for implementing this new type of healthcare can be considerable.

Physical exercise for depression was cost-effective from a 3-month healthcare provider perspective, but at a somewhat higher cost per QALY than ICBT. This was driven by higher intervention costs for physical exercise compared with ICBT, and slightly higher costs for use of other healthcare resources. Nevertheless, physical exercise was cost-effective compared with TAU when using the threshold of a WTP of €21 536, which has been suggested by the National Institute of Health and Care Excellence. This indicates that physical exercise is a promising treatment option and should be considered as an alternative to ICBT in the treatment of depression, e.g. for patients who prefer physical exercise over ICBT. The physical exercise sessions do not have to be given directly in primary care, but some kind of structured encouragement and adherence monitoring seems to be crucial. How this is to be administered and scheduled could be different in different country settings because of what is and is not included in their healthcare systems.

An important venue for future research is to investigate if ICBT and physical exercise could be altered to produce a larger effect on indirect costs, e.g. costs of sick leave and unemployment, which would make ICBT and physical exercise increasingly cost-effective also from a societal perspective.

In summary, ICBT for depression is probably cost-effective from a 3-month healthcare provider perspective as it leads to clinical improvements at acceptable additional costs. Physical exercise is also cost-effective from a 3-month healthcare provider perspective, if using conventional WTP thresholds. Implementing ICBT in primary care is a key for increasing accessibility to effective psychological treatment for depression, and monitored physical exercise should be considered as an alternative.
